# Specific Receptor Usage in *Plasmodium falciparum* Cytoadherence Is Associated with Disease Outcome

**DOI:** 10.1371/journal.pone.0014741

**Published:** 2011-03-03

**Authors:** Lucy B. Ochola, Bethsheba R. Siddondo, Harold Ocholla, Siana Nkya, Eva N. Kimani, Thomas N. Williams, Johnstone O. Makale, Anne Liljander, Britta C. Urban, Pete C. Bull, Tadge Szestak, Kevin Marsh, Alister G. Craig

**Affiliations:** 1 KEMRI/Wellcome Trust Research Programme, Centre for Geographic Medicine Research, Kilifi, Kenya; 2 Department of Molecular Parasitology, Liverpool School of Tropical Medicine, Liverpool, United Kingdom; 3 Muhimbili University of Health and Allied Sciences, Dar es Salaam, Tanzania; 4 Department of Medicine, Solna, Karolinska Institutet, Stockholm, Sweden; 5 Nuffield Department of Clinical Medicine, John Radcliffe Hospital, University of Oxford, Oxford, United Kingdom; 6 Department of Paediatrics, University of Oxford, Oxford, United Kingdom; London School of Hygiene and Tropical Medicine, United Kingdom

## Abstract

Our understanding of the basis of severe disease in malaria is incomplete. It is clear that pathology is in part related to the pro-inflammatory nature of the host response but a number of other factors are also thought to be involved, including the interaction between infected erythrocytes and endothelium. This is a complex system involving several host receptors and a major parasite-derived variant antigen (PfEMP1) expressed on the surface of the infected erythrocyte membrane. Previous studies have suggested a role for ICAM-1 in the pathology of cerebral malaria, although these have been inconclusive. In this study we have examined the cytoadherence patterns of 101 patient isolates from varying clinical syndromes to CD36 and ICAM-1, and have used variant ICAM-1 proteins to further characterise this adhesive phenotype. Our results show that increased binding to CD36 is associated with uncomplicated malaria while ICAM-1 adhesion is raised in parasites from cerebral malaria cases.

## Introduction

Infections by the human malaria parasite *Plasmodium falciparum* can lead to a range of severe outcomes including severe anaemia, organ failure, coma and eventually death. The sequestration of *P. falciparum* infected erythrocytes deep in micro-vasculature is implicated in malaria pathology [1]–[3]. To date 11 host receptors have been identified that mediate adhesion to endothelium including CD36 and ICAM-1 to which most clinical *P. falciparum* isolates adhere [4]. Binding to the endothelial receptor ICAM-1 has been associated with sequestration in brain vasculature [5] and showed a weak but not statistically significant association in one study with cerebral malaria (CM), while adhesion to CD36 showed no association [6]. Other adhesive phenotypes such as formation of rosettes [7]–[9] and the ability to bind receptors expressed on the placenta [10]–[12] have consistently shown strong associations with pathology. The parasite ligand responsible for binding, PfEMP1 (transcribed from the *var gene* family), contains motifs responsible for adhesion (Duffy Binding Like domains and cystein-rich interdomain regions [13]–[20] to a range of host receptors including ICAM-1 [21]; CD36[22], [23] P-Selectin [24]; PECAM/CD31 [25]; CR1 [13]. As demonstrated in the field of pregnancy associated malaria, characterizing the molecular nature of these interactions is important in understanding the pathogenesis of the more severe forms of malaria, because it would potentially provide more information in the design of new methods of prevention and treatment [10], [26]–[29].

Intercellular adhesion molecule 1 (ICAM-1) is a 90–115 kDa transmembrane glycoprotein expressed on a variety of cell types including endothelial cells [30]. Its expression *in vivo* is upregulated in response to a variety of inflammatory mediators including tumour necrosis factor (TNF) and interleukin-1 (IL-1). ICAM-1 is of interest because its expression on brain endothelium is up-regulated in severe malaria [1] and a natural polymorphism termed ICAM-1^Kilifi^ affects disease severity in some parts of Africa [31], [32]. This mutation involves a lysine to methionine substitution at position 29 and is present at a gene frequency of 30% in African populations [31]–[33]. Binding to ICAM-1 is not a simple phenotype and a number of laboratory-adapted parasite lines such as A4u, ItG, C24 and JDP8, show varying binding phenotypes under flow or static conditions. Tse and others in 2004, targeted amino acid residues within the ICAM-1 protein and introduced alanine mutations that resulted in 25 mutant proteins, including ICAM-1^S22/A^, in which serine at position 22 is replaced by alanine. This and earlier studies [34], [35] revealed that A4u and ItG demonstrated differential binding abilities to ICAM-1^Reference^, ICAM-1^Kilifi^ and ICAM-1^S22/A^. The laboratory line A4u binds moderately to ICAM-1 [36] with little stationary adhesion under flow whereas the main adhesive interactions were of the rolling type. ItG and JDP8 showed stronger stationary binding but significantly lower rolling under these conditions [34]–[37]. The laboratory line C24 on the other hand does not bind to ICAM-1. A4u, ItG and C24 bound to CD36 while JDP8 showed significantly lower adhesion to this receptor. These variations in binding may be explained by the differential adhesion properties of the variant surface proteins (PfEMP1) expressed by each parasite line or the expression levels of PfEMP1.

CD36 is an 88 kDa integral membrane protein found on various host cells including endothelium, monocytes and platelets. This receptor has multiple functions that include platelet adhesion, non-opsonic phagocytic clearance of senescent or pathologically altered cellular structures by pattern recognition [38] and regulation of different metabolic pathways, membrane transport systems and immune responses in humans [39], [40]. Almost all *P. falciparum* paediatric clinical isolates adhere to CD36 and no difference in CD36-binding of parasites from severe or uncomplicated parasites has been observed [6], [41]. However, a recent paper has suggested that CD36 deficiency may protect against *falciparum* malarial anaemia [42] and pregnancy-associated *P. falciparum* isolates were found not to bind to CD36 [10], [43].

Adhesion of *P. falciparum* laboratory lines or clinical isolates has been assessed using two techniques, namely static and laminar flow adhesion assays. The former measures immobilization of parasitized cells on proteins, while the latter attempts to mimic the physiological system allowing for measurements of the strength or avidity of the interaction between infected cells and endothelial receptors [44], [45]. The majority of adhesion studies conducted so far have concentrated on adhesion of clinical isolates under static conditions, while here we consider the effect of flow and static conditions in parallel to give different information about ligand-receptor interactions. For example studies under static conditions, using ItG and JDP8 revealed that these parasites are high avidity ICAM-1 binders, when presented on TNF-activated HUVEC [36], but under laminar flow these parasites bound poorly. A4u showed the opposite effect to that observed with ItG and JDP8 and bound strongly under flow conditions. Our intention in using both types of assays for ICAM-1 binding is to take into account previous work with this receptor indicating that its role in adhesion is particularly associated with recruitment from flow [45].

Genetic polymorphisms of affected populations and the presence of specific falciparum gentoypes can both modulate the impact of *falciparum* malaria infections in endemic areas. Mutations in the human genes ICAM-1, CD36 and globin genes have been associated with susceptibility to severe *falciparum* malaria [46]–[48], in some but not all populations. For example, the homozygous ICAM-1^Kilifi^ has previously been shown to be associated with cerebral malaria in Kenya, while in Malawi, children with the same phenotype showed increased mortality (Turner, unpublished observations). Other studies in West Africa did not show any association [33] and a more recent study in Kilifi suggested that the effect of the ICAM-1^Kilifi^ mutation is probably more important in infections other than malaria [48]. In addition, children homozyogous for a mutation change of T to G in the CD36 gene at nucleotide position 188 in exon 10 [49] are protected against severe malarial anaemia and children carrying the sickle cell trait, HbS are protected against all forms of malaria disease [42], [46], [47], [50]–[52].

We have analysed the role of polymorphic variants of ICAM-1 in severe *falciparum* malaria using static and flow based adhesion assays and CD36 in static assays only. Understanding the adhesion mechanisms of parasites will provide us with a better understanding of disease progression.

## Materials and Methods

### Ethics Statement

The proposal for this study was reviewed and passed as ethically acceptable by the Kenya Medical Research Institute and National Ethic Review Board and the Oxford Tropical Medicine Research Ethics Committee (OXTREC). In addition, a fully informed written consent was obtained from parents/legal guardians of every child enrolled in the study.

### Collection and culture of *P. falciparum* clinical isolates

Parasite samples were collected between 2005 and 2009 from children attending Kilifi District Hospital, Kenya who had a primary diagnosis of *P. falciparum* malaria and from a cohort of children under active surveillance for acute malaria in the Junju area of Kilifi District in Kenya. The samples were collected before anti-malarial treatment was administered. Only children with a parasitaemia above 3% were included in the study as this level of parasitaemia is required for the adhesion assays. Taking advantage of an already established algorithm [53] that allocates a unique, anonymous identifier for selection and processing of patient blood samples, patients were chosen using the inclusion criteria shown below. Sample processing, adhesion assays and data analysis were performed using the unique identifier so that the origin and clinical details of the sample remained unknown until the study was completed. Children presenting with febrile illness and peripheral *P. falciparum* parasitaemia were included in this study.

Severe malaria defined as hospital admissions, was further divided into three categories namely: Cerebral Malaria (CM) with a Blantyre Coma Score (BCS) of ≤2 [54], [55], Severe malaria anaemia (SMA) had a haemoglobin concentration <5g per deciliter, [56] and Severe malaria-other (SM-other) patients with neither severe anaemia nor cerebral malaria (BCS>2). Lastly, patients recruited from peripheral dispensaries with no severe complications were termed Non-severe malaria (Uncomplicated, UM) [55]–[58].

167 patient samples were entered into the study, consisting of 85 frozen and 82 fresh isolates. 66 frozen isolates failed to meet the requirements of the adhesion assays (either through failure to grow or insufficient material recovered for the binding assays) giving a total of 101 isolates that were investigated for their binding to ICAM-1 and CD36.

### Culture of parasites

*P. falciparum* lines derived from the laboratory line IT4/25/5 [59], [60], ITO4-A4u (A4u) and ItG, which have previously been selected for binding on ICAM-1, were used as controls. To minimize the effect of antigenic switching in culture, a batch of stabilates, selected on a variant specific monoclonal antibody BC6 (A4u) and by repeated selection on ICAM-1 (ItG) were serially thawed and maintained in culture for not more than three weeks.

Parasites were prepared as previously described [58] from acute samples. In brief, RBCs were purified from mononuclear cells and granulocytes using lymphoprep and Plasmion, respectively. Parasites were grown to maturity for 24 to 48 hours either from frozen (n = 19) or fresh (n = 82) isolates.

Laboratory parasite lines and parasites from patient isolates were cultured from pre-treatment blood samples at 2% hematocrit in O^+^ human erythrocytes using standard culturing techniques, [61] using RPMI 1640 medium (supplemented with 37.5 mM HEPES, 7 mM D-glucose, 6 mM NaOH, 25 µg of gentamicin sulfate/ml, 2 mM L-glutamine, and 10% human serum) at a pH of 7.2 in a gas mixture of 96% nitrogen, 3% carbon dioxide, and 1% oxygen. The parasite density was calculated by determination of the number of parasites per 200 white blood cells (WBC) for thick blood film or 500 red blood cells (RBC) for thin blood films. Baseline WBC or RBC counts were used to calculate the parasitaemia (parasites/µl). For all adhesion assays the parasitaemia was adjusted to 3% and 1% hematocrit (approximately 30000 parasites/µl, based on an estimation of 10^9^ RBCs/100µl).

### Recombinant proteins

Purified receptors used in this study were CD36 (R & D Systems, UK), ICAM-1-Fc reference (the wild type allele of ICAM-1 (ICAM-1^Reference^)) [37], ICAM-1 Kilifi (ICAM-1^Kilifi^) [16], [38] and ICAM-1 S22/A (ICAM-1^S22/A^) [62].

### Static adhesion assays

Purified recombinant proteins were spotted in triplicate in a radial pattern using 2 µl spots on 60×15 mm bacteriological plastic petri dishes (Falcon 1007; Becton Dickinson, Oxford, UK) at concentrations of 50 µg/ml for ICAM-1 and CD36 proteins. These concentrations had previously been shown to be within the dynamic range for detecting differences in adhesion and produce coated surfaces with receptors at levels approximately equal to receptor densities seen on activated endothelium or basal levels of CD36 [36]. The dishes were placed in a humidified chamber for 2hrs at 37°C to allow the proteins to adsorb to the surface, after which the protein solutions were aspirated off and the uncoated plastic area blocked overnight with 1% BSA/PBS at 4°C. The plates were warmed at 37°C for one hour, blocking solution (1% BSA/PBS) removed and washed twice in binding buffer (RPMI 1640 in 2% glucose) prior to adding 1.25 ml of parasite cultures at 3% parasitaemia, which had been washed twice in binding buffer and resuspended to 1% haematocrit. The plates were incubated at 37°C for one hour with gentle resuspension every 10 minutes. Unbound infected and uninfected erythrocytes were removed by gentle manual washing (4–6 washes) with 1.5 ml binding medium (monitoring of adhered cells was performed using an inverted microscope). The adhered infected erythrocytes were fixed with 1% glutaraldehyde in phosphate buffered saline for 1 hour and stained with 10% giemsa for 15 minutes. Adhesion levels were quantified by microscopy using the unique, anonymous identifier (with the operator blinded to the clinical category) and results were expressed as the mean number of parasitized erythrocytes bound per mm^2^ of surface area.

### Flow adhesion assays

Using a modified method [36], [44] microslides (Camlab, UK) were coated with ICAM-1 recombinant proteins at 50 µg/ml at 4°C overnight followed by blocking with 1% BSA/PBS at 37°C for two hours. Microslides were connected to tubing mounted on a microscope stage (Nikon; Eclipse TE 200) enclosed within a plastic chamber that maintains the temperature at 37°C. Tubing was mounted to a system that allows medium to flow through at a controlled rate of 186 µl/minute (0.05 Pa). Infected erythrocyte suspensions were passed over the slide for a total of 6 minutes followed by buffer flowed to remove any unbound cells (2 minutes). This flow rate mimics the flow rate in the micro-vasculature.

At the end of each 8 minute run, the total number of stationary infected erythrocytes was counted in six separate fields on each microslide with the buffer still running. Using one protein per microslide, all controls and clinical samples were run in duplicate in a single experiment.

### Patient and parasite genotypes

CD36, ICAM-1, α-thalassemia and sickle cell genotypes were determined in all patients using allele-specific polymerase chain reaction described elsewhere [31], [33], [49], [63], [64]. To determine the extent of mixed parasite populations a multi-plexed fluorescent labeling system for genotyping MSP1 (subfamilies MAD20, K1, RO33) and MSP2 (subfamilies FC27 and IC) was used [65].

### Binding signatures for *P. falciparum* clinical isolates under static conditions

The larger dataset for static assays was available for further analysis of the ICAM-1-binding phenotype. The binding data of clinical isolates was converted into binding signatures by comparing their ICAM-1^Kilifi^ and ICAM-1^S22/A^ adhesion against that of ICAM-1^Reference^ as the reference standard. This allowed us to subtype these isolates for ICAM-1 adhesion using a two-letter code based on relative binding to Kilifi and S22/A proteins respectively. The absolute binding values obtained were converted into percentage values relative to ICAM-1^Reference^ binding and designated high binders 80–100% (binding signature ‘a’), moderate binders 50–79% (binding signature ‘b’) and low binders 0–49% (binding signature ‘c’) (see Table S1).

### Data Analysis

Statistical analysis was carried out using STATA (version 9.0) and Prism (version 5.0). Distributions were frequently highly skewed from normal so non-parametric tests of significance (e.g. Mann-Whitney) were applied.

## Results

### Study Population

**Figure 1:**
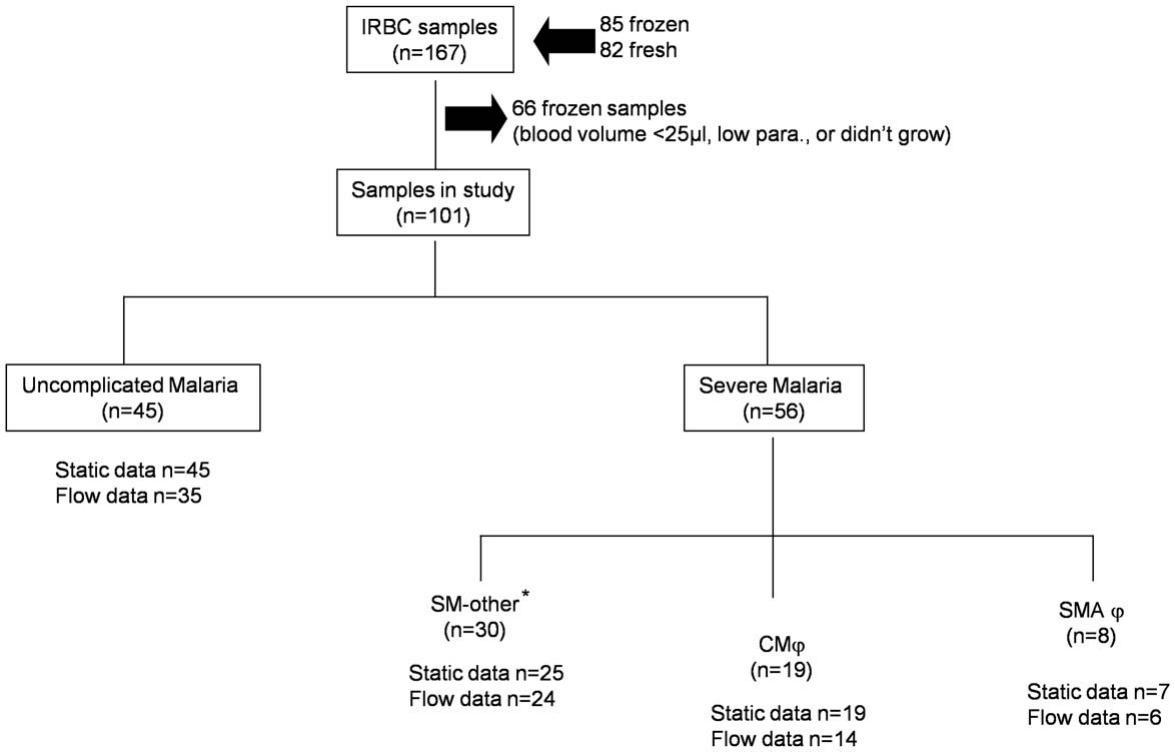
Distribution of clinical samples used in the study. Scheme showing the collection and classification of infected blood samples from children with different malaria syndromes: uncomplicated (UM), severe malaria other (SM other); cerebral malaria (CM); severe malaria anaemia (SMA). The following symbols designate: * severe malaria but no CM or anaemia; ϕ one patient had an Hb<5g/dl and CM; n = number of clinical samples; low para, low parasitaemia

From a total of 167 blood samples screened for parasite growth and recovery only 101 were used for adhesion studies. Previous studies used frozen ring stage parasites for adhesion assays [6], but in our study, these frequently failed to grow or did not achieve the required parasitaemia of ≥30,000/µl required for accurate assessment of binding to different ICAM-1 variants (Figure 1). In addition, other studies [41], [66], including some recently published data [67], have suggested that significant phenotypic changes in *var* expression may take place on freezing and thawing particularly if parasite cultures are grown *in vitro* for 5–6 cycles. Isolates in this study were grown for a single cycle and because most frozen isolates failed to grow (only 19 out of a total of 66 attempted) we resorted to using fresh isolates (n = 82), to give a total of 101 isolates that were analysed for adhesion (Figure 1). The growth characteristics for isolates from both UM and severe malaria cases were similar.

Out of the 101 children, 45 had uncomplicated malaria, while 56 had severe malaria. Children with severe disease (SMA, CM and severe malaria-other) had a median age of 4 years (interquartile range 3–6 years) and those with uncomplicated malaria had a median age of 5 years (interquartile range 4–6 years). All children were free of other secondary infections and were split into different clinical groups as shown in Table 1. We observed that children with severe malaria-other had a high median parasitaemia of 582120 parasites/µl (interquartile range 194140–739405), followed by children with CM (median 457420, interquartile range 230880–630450) and those with SMA and uncomplicated malaria had a lower median parasitaemia of 228000 and 235000, respectively (Table 1). The parasites that grew to trophozoite stage were assessed for adhesion under static and flow conditions, although in some cases the sample volume was limited and static adhesion alone was carried out (Figure 1). Cases of SMA were infrequent in this population (n = 8) and only one patient had both cerebral malaria and a haemoglobin (Hb) of less than 5 g/dl. This patient was considered in both the CM and SMA arm.

**Table 1:** Clinical samples included in the study and classified into different severity groups.

Severity group	Clinical status	n	Parasite count (per/µl)	Median age (yr)
**Severe anaemia ϕ**	Hb<5 g/dl	8	228000 (166870–255900)	2.5 (1.8–3.3)
**Cerebral malariaϕ**	BCS≤2, prostrated unconscious	19	457420 (230880–630450)	3 (2–4)
**Severe malaria-other** *	BCS>2	30	582120 (194140–739405)	3 (2–5)
**Uncomplicated malaria**	Mild disease, no severe complications	45	235000 (136300–385400)	5 (4–6)

Numbers in parentheses represent the interquartile ranges.

*Severe malaria-other-severely ill children who did not meet the criteria for severe anemia or cerebral malaria.

ϕ 1 patient had both CM and anaemia.

### Effect of clinical syndromes on adhesion

**Figure 2:**
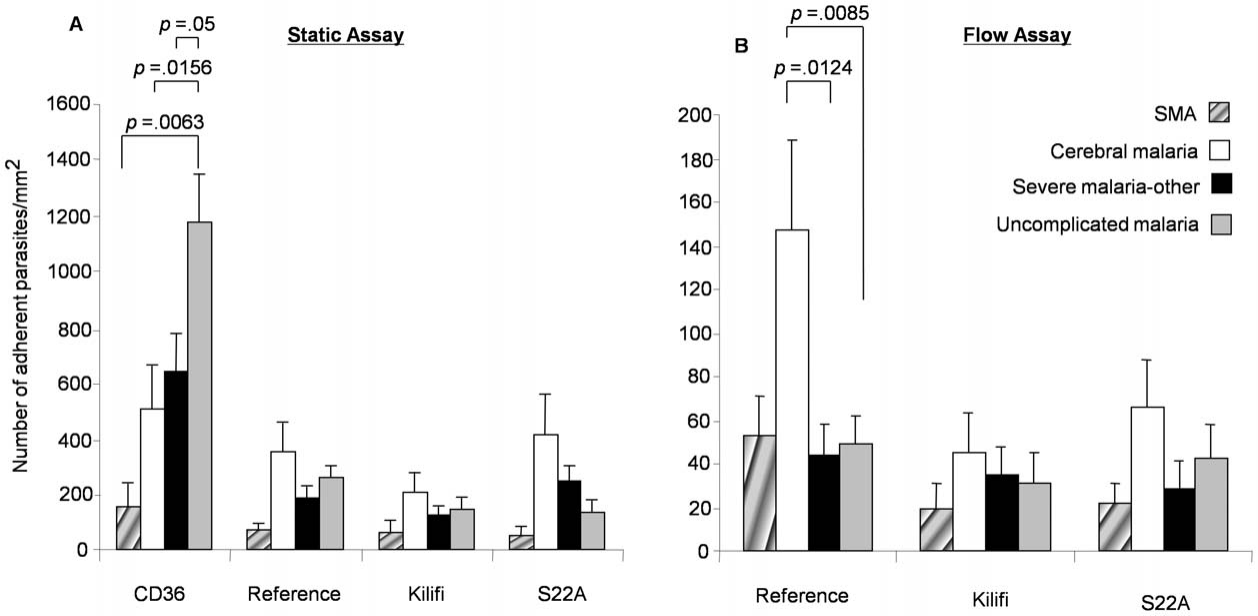
Mean adhesion of clinical isolates to CD36 and ICAM-1 proteins. Experiments were performed under static (a) and flow (b) conditions at coating concentrations for CD36 and ICAM-1 proteins of 50 µg/ml. Data represent the mean number of adherent parasites per mm^2^±standard error. p values that describe significant associations are shown.

In this study, we observed that 80% of clinical isolates bound to both CD36 and ICAM-1 (Table S2). As with previous findings [6], children with an Hb of <5 g/dl showed reduced adhesion under static conditions (Figure 2a). For static conditions, we have shown significantly higher binding to CD36 by UM isolates [mean adhesion 1177.2 parasites/mm^2^ (95% confidence interval (CI), 829.0–1525.3)] compared to CM isolates [mean adhesion 502.3 parasites/mm^2^ (95% CI, 179.6–825.0) (p = 0.016, Mann-Whitney test)] and SMA isolates [mean adhesion 158.5 (95% CI -38.4–355.4) (p = 0.006, Mann-Whitney test)] . Intermediate significance was observed when UM cases were compared to children with SM-other [mean adhesion 645.0 (95%CI, 370.6–919.4)] to CD36 under static conditions (p = 0.050, Mann-Whitney test). Children below one year admitted with severe disease, irrespective of the clinical syndrome, showed reduced levels of adhesion to all three ICAM-1 protein variants and CD36 under static and flow conditions, but numbers were too few (n = 8) to test for significance.

We did not observe differences in binding densities to ICAM-1 protein variants under stationary adhesion conditions. Under flow conditions, however, CM cases (accounting for 30% of total number of children recruited) showed the highest adhesion under flow conditions. Mean adhesion under flow to ICAM-1^Reference^ for CM was 158.0 parasites/mm^2^ (95% CI, 63.6–252.4), for SMA 52.8 parasites/mm^2^ (95% CI, −8.7–114.3), SM-other 42.8 parasites/mm^2^ (95% CI, 13.0–72.6) and UM 48.7 parasites/mm^2^ (95% CI, 21.9–75.5) (Figure 2b). Binding densities to ICAM-1^Reference^ were significantly higher for CM isolates than for isolates from children with UM or SM-other (Figure 2b), under flow conditions (p = 0.009 and 0.012 respectively, Mann-Whitney).

### Association of host or parasite genotypes with adhesion

There was no significant difference in the mean cytoadherence values or clinical syndromes between isolates from patients with different α-Thalassemia, ICAM-1, CD36 genotypes and HbS, sickle cell genotypes (Table S3) or between patients with isolates with different MSP1/ MSP2 genotypes or numbers of circulating genotypes. The majority of children had 2 circulating parasite genotypes and the mean number of parasite genotypes was greatest in CM cases and lowest in UM cases, although these differences were not statistically significant (Table 2). Among the 101 patients screened, we found that all were HbAA (normal haemoglobin) except for three UM cases that were HbAS, sickle trait carriers.

**Table 2:** Association of clinical syndromes with MSP1 and MSP2 parasite genotypes.

	Alleles	CM	SMA	SM-O	UM
**MSP1**	MAD20	14.5	15.4	10.6	9.5
	K1	27.1	15.4	31.8	28.6
	RO33	10.4	23.1	7.6	23.8
**MSP2**	FC27	16.7	15.4	24.2	23.8
	IC	31.3	30.8	25.8	14.2
**Mean no. of genotypes (95% CI)**		2.9 (2.5–3.2)	2.2 (1.5–2.8)	2.6 (2.1–3.0)	2.6 (1.5–3.7)

Data is presented as a % of respective genotypes in each clinical category.

CM, cerebral malaria; SMA, severe malarial anemia; UM, uncomplicated malaria; SM-O, severe malaria-other-severely ill children who did not meet the criteria for severe anemia, cerebral malaria.

### Analysis of ICAM-1 binding signatures

**Figure 3:**
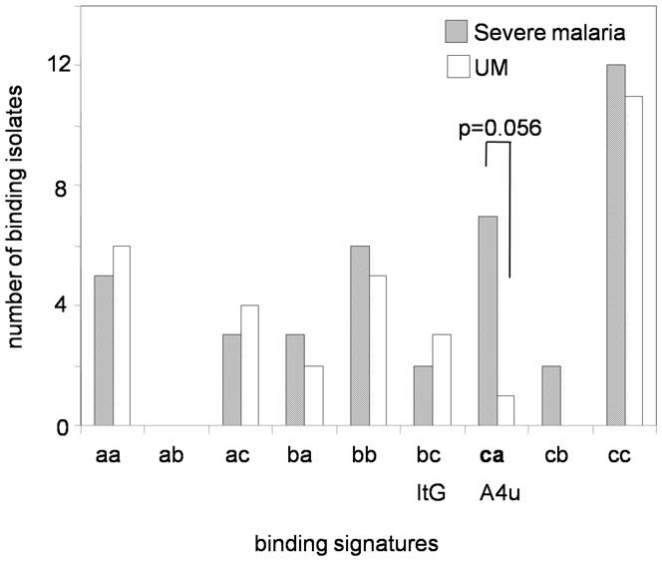
Binding signatures for *P. falciparum* clinical isolates under static conditions. Binding signatures calculated by comparing ICAM-1^Kilifi^ and ICAM-1^S22/A^ clinical binding data against that of ICAM-1^Reference^ the reference standard. Data was calculated using adhesion to ICAM-1^Reference^ as a standard against which relative adhesion to ICAM-1 ^Kilifi^ and ^S22/A^ are calculated. a, designates strong (80–100%), b, moderate (50–79%) and c, low (0–49%) adhesion. The number of isolates with Severe malaria n = 35 and UM n = 32.

Since a weak association between ICAM-1 binding and pathology has been suggested by previous work, further analysis of the binding phenotypes of clinical isolates for adhesion to ICAM-1 variant proteins was performed to develop a more detailed picture of the nature of this interaction (Figure 3 and Table S1). Clinical isolates were found to exhibit differential binding densities to the three ICAM-1 protein variants Reference, Kilifi and S22/A. This allowed us to sub-type the binding to ICAM-1 into different categories, for example, A4u binds weakly to ICAM-1^Kilifi^ and strongly to ICAM-1^S22/A^ and when these were compared against ICAM-1^Reference^ it gives a ‘ca’ type (c = low binding; a = strong binding). ItG on the other hand, gives a ‘bc’ type (b = moderate binding to Kilifi and c = low binding to S22/A when compared to Reference). Similarly clinical isolates display varying binding signatures when ICAM-1^Reference^ is taken as the reference point (Table S1). Data analysis was performed using static data only. The number of isolates in Severe Malaria-other were n = 22, CM n = 13 and UM n = 32. Isolates that gave no adhesion to ICAM-1 proteins were excluded from this analysis. Of the nine ICAM-1 binding signatures defined by this approach, only the “ca” type was more frequent among the severe malaria isolates than among the UM isolates, but this was not statistically significant (Odds Ratio 7.750 (95%CI 0.896–67.02) p = 0.056, two-tailed Fishers exact).

## Discussion

This current study assessed the adhesion of *P. falciparum* infected erythrocytes under flow (ICAM-1) and static (ICAM-1 and CD36) conditions from children with different clinical syndromes of malaria. *P. falciparum* isolates from children with UM have significantly higher levels of adhesion to CD36, under static conditions, than isolates from children with severe disease (CM, Severe Malaria-other and SMA). Previous studies had revealed that almost all isolates of *P. falciparum* adhere to CD36 [2], [42], [68], [69] and in Africa no differences in CD36-binding ability between SM and UM cases were observed [6], [25], [41], [70], although one study in Mali showed a positive association with a sub-type of PfEMP1 (*cys2var*) and severe malaria and this *var* type is part of a group of PfEMP1 proteins that tend to have lower binding to CD36 [71]. In adults in South East Asia, higher CD36 binding (using C32 cells) was observed among *P. falciparum* isolates from patients with severe (but not cerebral) malaria [72]. Most of these earlier studies used frozen parasite isolates and the sample sizes were relatively small. In our study, freshly grown parasite isolates have given some different patterns of association between binding and clinical phenotypes than the frozen ones used in the Newbold *et al* paper [6], although we did not have an asymptomatic group which was present in this latter study so care needs to be taken in making this comparison.

One potential weakness of this study is that bias may have been introduced by only analyzing parasites isolates with an *in vivo* parasitaemia that exceeds 30000/µl, but for adhesion based studies such parasitaemias are required to obtain accurate measurements. It is possible that this cut off may have resulted in the exclusion of SMA cases which often have parasitaemia below 30000/µl [73], [74]. However this parasitaemia may also be an advantage in that the UM controls will be from patients with relatively high parasitaemia and so better matched to the CM isolates. A second potential weakness is that adhesion to CD36 was not studied under laminar flow conditions as sample volumes were limiting, reducing the number of analyses possible. Data relating to *var* types of patients is not currently available but this work is in progress and forms part of a large study examining many factors of parasite biology (P. Bull, personal communication). This information once available may explain why we observed reduced adhesion in children below one year of age, based on the subset of PfEMP1 proteins observed in this category.

CD36 receptor is involved in non-opsonic phagocytosis of parasite infected erythrocytes by monocytes and macrophages [38], [75], [76] and in the uptake of antigens into dendritic cells, (DCs) [77], [78]. Binding of infected erythrocytes to DCs modulates their maturation in vitro [76] leading to upregulation of IL-10 and down regulation of IL-12. This may in turn limit the production of inflammatory responses that are associated with severe clinical outcomes. The high levels of adhesion of UM cases to CD36 observed in this study suggest a role of this receptor in host control of parasitaemia, before antigen-specific immune responses occur and/or limitation of pro-inflammatory responses. This hypothesis is in contrast to the common view of CD36 as a receptor exploited by the parasite for the purpose of tissue sequestration.

The pathogenesis of severe *falciparum* malarial anaemia arises due to decreased production and increased destruction of erythrocytes [79], [80]. Unlike high malaria transmission areas where severe malaria anaemia cases are common [81], [82], the incidence of SMA has fallen dramatically in Kilifi district over the last ten years [83]. The reduction in adhesion observed in the SMA group was not due to the presence of polymorphisms such as sickle cell, α-thalassemia or CD36-deficiency as previously shown by other groups [42], [51], [84]. A modification in the surface membrane of erythrocytes leading to alterations in expression of surface host receptors CR1, CD55 and CD59 has previously been shown to occur in erythrocytes from SMA children [85], whether this affects PfEMP1 display remains to be determined but this could explain why isolates from these patients show reduced adhesion [30]. Furthermore, four SMA out of the eight were microcytic with a mean corpuscular volume (MCV) below normal range (data not shown) and one of these patients had respiratory distress. Despite MCV being below normal range, this did not explain reduced adhesion as values varied when compared between patients independent of MCV. It is also possible that SMA may lead to the selection of particular PfEMP1 variants, as seen in children with Hb-S and Hb-C, which could affect their adhesion characteristics (Fairhurst, pers. comm.). Further studies are needed to understand the molecular basis of this phenomenon.

Detectable binding of *P. falciparum* clinical isolates to ICAM-1 had previously been shown to have a prevalence of 80% in this population [6] and our study found similar levels (Table S2). Newbold and others [6] did not observe a significant association with CM but with malaria disease (CM and UM combined) when compared with asymptomatic infection. We found that binding to ICAM-1^Reference^ is associated with CM, and levels of binding of isolates from patients with this clinical syndrome were found to be significantly higher than UM cases when using flow-based assays. Under flow, but not static conditions, ICAM-1 captures infected erythrocytes, suggesting that this process of capturing from flow is a critical one in the pathogenesis of CM. This is in agreement with previous findings that in some laboratory isolates ICAM-1 is important for efficient adhesion to HUVEC [36] and suggests a mechanism for severe disease based on preferential recruitment of specific adhesive types of parasites in the circulation to brain endothelium. This would also fit with other work, which showed that the adhesion of different parasite variants was not evenly distributed among tissues, but instead that accumulation of parasites expressing particular *var*-types was organ-specific (including brain) [86].

ICAM-1 is also an attachment molecule for the major serotypes of human rhinovirus (HRV) that are responsible for localizing the virus near the cellular membrane and triggering conformational changes in the viral capsid that initiate uncoating and epithelial cell invasion [87]. Studies on human rhinovirus (HRV) using two different HRV serotypes have shown varying adhesion phenotypes to ICAM-1^Reference^ and ICAM-1^Kilifi^, and their association with varying clinical outcome [88]. A similar situation may occur in *P. falciparum* variants from the field as shown in this study and in laboratory lines [63] that exhibit differential adhesion abilities to all three ICAM-1 proteins. When we tested patient isolates for their ability to bind to three different forms of ICAM-1 under static conditions, we saw a broad distribution of adhesion types but an excess of ‘ca’ type in severe malaria (CM and SM other combined, although the majority of ‘ca’ types were in the CM category (Table S1)). It should be noted that this association did not reach statistical significance (p = 0.056). It is possible that this binding signature is linked to pathogenesis, although further studies are required to study this. The ‘ca’ binding signature is shared with the A4 parasite variant, and the ITO4 line, which was isolated after selection on activated human umbilical vein endothelial cells (HUVEC). In contrast, ItG parasites selected by binding to purified ICAM-1 protein displayed the ‘bc’ binding signature, which was not prevalent among parasites isolated from children with severe malaria (Figure 3). HUVEC expresses ICAM-1 on TNF stimulation but not CD36 and is used widely as a model of brain sequestration *in vitro* [21], [36], [59], [89]. Thus a binding signature exhibited by parasites isolated from children with severe malaria is similar to that exhibited by a parasite line selected for binding to endothelial cells with a receptor distribution similar to that seen in the brain. However, another parasite line from the IT parent line, ItG, is not associated with severe *falciparum* malaria despite being selected on ICAM-1, implying that the cellular context of the receptor may also be important. The numbers available for this part of the study were limited and further work will be required to confirm this observation.

The number of circulating genotypes in a given sample indicates the extent of parasite diversity and may explain varying adhesion phenotypes observed. This study analysed MSP1 families (MAD20, K1, RO33) and MSP2 families (FC27, IC) to answer this question. We did not see any associations with the number of or type of parasite genotypes and clinical syndromes. A limited parasite diversity as observed in a previous study [90] was seen. Conflicting results between the allelic family present and association with disease severity have been seen in other studies showing MSP2/ IC type dominant in asymptomatic infections [91], [92] while others showed an association of MSP2/ FC27 with symptomatic malaria [93]. Our study also observed the predominance of the allelic family MSP1/ K1 as seen in a study in Gabon [94]. Human genotypes like the haemoglobinopathies HbS and HbC are known to be relevant in cytoadherence. A recent study suggested that these polymorphisms can affect the display of PfEMP1 and thereby reduce adhesion [52]. In our study we found only three HbAS carriers, which was too few to draw any conclusions. We further found no evidence of an association between carriage of particular allelic families of msp1 or msp2 and either severity of clinical presentation, or adhesion phenotype.

As with pregnancy-associated malaria, the aim now is to use this information to understand the basis of severe malaria and identify novel approaches to prevention or treatment. This will not be a simple task (see [4] for a review of this area), due to several factors such as severe malaria consisting of a diverse collection of syndromes and the involvement of multiple receptors producing synergy such as that observed between ICAM-1 and CD36. Indeed the role of other receptors besides ICAM-1 and CD36 should not be discounted in terms of their contribution to disease, for example, gC1qR can act as a receptor for binding to brain microvascular endothelial cell lines [95] but its role in CM is yet to be determined.

In summary, this study has identified significant associations between receptor usage by pediatric clinical isolates and disease severity. We have shown for the first time in African children that parasites isolated from children with UM bind to CD36 at higher densities than parasites from children with severe malaria. Importantly, high density parasite binding to ICAM-1 under flow conditions was significantly associated with cerebral malaria. The findings presented here provide a platform from which to focus efforts on blocking or reversing ICAM-1-mediated adhesion to reduce cerebral malaria as well as a reconsideration of anti-CD36 strategies for controlling severe malaria.

## Supporting Information

Table S1 Binding signatures for clinical isolates under static conditions. Binding signatures of clinical isolates presented as % binding and coloured codes. K29, % adhesion to ICAM-1^Kilifi^, S22, % adhesion to ICAM-1^S22/A^ and column 3, binding signature. Binding is calculated relative to ICAM-1^Reference^ as reference standard. The numbers of isolates in SMA n = 5, CM n = 13, Severe Malaria-other n = 22 and UM n = 32.
(0.08 MB DOC)

Table S2 Adhesion data for clinical P. falciparum isolates under static and flow adhesion assays. Numbers represent number of adherent parasites/mm^2^. (-) no data available.
(0.14 MB DOC)

Table S3 Genotype frequencies for ICAM-1, CD36, α-thalassaemia and HbS. Genotype frequencies for patients studied. HbS, haemoglobin S variant (sickle cell genotype). CD36, point mutation from T to G in the CD36 gene at nucleotide position 188 in exon 10. Numbers represent the number of children that fall into each genotype (wild-type, heterozygous and homozygous). Numbers in parenthesis is given as a percentage of respective genotypes.
(0.03 MB DOC)
