# Supplementary material for: Specific Receptor Usage in Plasmodium falciparum Cytoadherence Is Associated with Disease Outcome
Source: PLoS One. 2011 Mar 3;6(3):e14741. doi: 10.1371/journal.pone.0014741 (PMC3048392; doi:10.1371/journal.pone.0014741)
Supplement: Table S1 — Binding signatures for clinical isolates under static conditions. Binding signatures of clinical isolates presented as % binding and coloured codes. K29, % adhesion to ICAM-1Kilifi, S22, % adhesion to ICAM-1S22/A and column 3, binding signature. Binding is calculated relative to ICAM-1Reference as reference standard. The numbers of isolates in SMA n = 5, CM n = 13, Severe Malaria-other n = 22 and UM n = 32. (0.08 MB DOC) [file pone.0014741.s001.doc]

**Table S1. Binding signatures for clinical isolates under static conditions**

| **SMA** | | | **CM** | | | **Severe malaria-other** | | | **UM** | | |
| --- | --- | --- | --- | --- | --- | --- | --- | --- | --- | --- | --- |
| **K29** | **S22** |  | **K29** | **S22** |  | **K29** | **S22** |  | **K29** | **S22** |  |
| 26 | 67 | cb | 63 | 60 | bb | 51 | 57 | bb | 67 | 56 | bb |
| 77 | 62 | bb | 14 | 99 | **ca** | 31 | 16 | cc | 69 | 79 | bb |
| 116 | 126 | aa | 26 | 89 | **ca** | 117 | 92 | aa | 101 | 100 | aa |
| 561 | 5 | ac | 61 | 108 | ba | 44 | 42 | cc | 114 | 34 | ac |
| 1 | 48 | cc | 313 | 246 | aa | 56 | 52 | bb | 103 | 27 | ac |
|  |  |  | 59 | 12 | bc | 42 | 69 | cb | 74 | 75 | bb |
|  |  |  | 76 | 36 | bc | 94 | 36 | ac | 104 | 95 | aa |
|  |  |  | 4 | 0 | cc | 2 | 5 | cc | 81 | 37 | ac |
|  |  |  | 0 | 170 | **ca** | 4 | 39 | cc | 93 | 84 | aa |
|  |  |  | 69 | 177 | ba | 89 | 86 | aa | 0 | 0 | cc |
|  |  |  | 64 | 165 | ba | 31 | 0 | cc | 0 | 0 | cc |
|  |  |  | 27 | 0 | cc | 49 | 194 | **ca** | 21 | 743 | **ca** |
|  |  |  | 11 | 120 | **ca** | 0 | 0 | cc | 58 | 50 | bb |
|  |  |  |  |  |  | 71 | 69 | bb | 56 | 28 | bc |
|  |  |  |  |  |  | 124 | 9 | ac | 70 | 148 | ba |
|  |  |  |  |  |  | 98 | 126 | aa | 0 | 0 | cc |
|  |  |  |  |  |  | 0 | 0 | cc | 0 | 0 | cc |
|  |  |  |  |  |  | 22 | 95 | **ca** | 65 | 84 | ba |
|  |  |  |  |  |  | 0 | 394 | **ca** | 99 | 90 | aa |
|  |  |  |  |  |  | 78 | 52 | bb | 106 | 98 | aa |
|  |  |  |  |  |  | 0 | 23 | cc | 22 | 9 | cc |
|  |  |  |  |  |  |  |  |  | 0 | 0 | cc |
|  |  |  |  |  |  |  |  |  | 0 | 0 | cc |
|  |  |  |  |  |  |  |  |  | 0 | 0 | cc |
|  |  |  |  |  |  |  |  |  | 15 | 2 | cc |
|  |  |  |  |  |  |  |  |  | 30 | 15 | cc |
|  |  |  |  |  |  |  |  |  | 34 | 8 | cc |
|  |  |  |  |  |  |  |  |  | 50 | 57 | bb |
|  |  |  |  |  |  |  |  |  | 89 | 0 | ac |

Binding signatures of clinical isolates presented as % binding and coloured codes. **K29,** % adhesion to ICAM-1^Kilifi^, **S22**, % adhesion to ICAM-1 ^S22/A^ and **column** **3**, binding signature. Binding is calculated relative to ICAM-1^Reference^ as reference standard. The numbers of isolates in SMA n=5, CM n= 13, Severe Malaria-other n=22 and UM n=32.

Colours denote 80-100% , 50-79% , 0-49% adhesion
