# Supplementary material for: Specific Receptor Usage in Plasmodium falciparum Cytoadherence Is Associated with Disease Outcome
Source: PLoS One. 2011 Mar 3;6(3):e14741. doi: 10.1371/journal.pone.0014741 (PMC3048392; doi:10.1371/journal.pone.0014741)
Supplement: Table S2 — Adhesion data for clinical P. falciparum isolates under static and flow adhesion assays. Numbers represent number of adherent parasites/mm2. (-) no data available. (0.14 MB DOC) [file pone.0014741.s002.doc]

**Table S2. Adhesion data for clinical *P. falciparum* isolates under static and flow adhesion assays.**

| **Severity**  **Group** |  | **Static Adhesion assay** | | | | **Flow adhesion assay** | | |
| --- | --- | --- | --- | --- | --- | --- | --- | --- |
|  | **Patient No** | **CD36** | **Ref** | **Kilifi** | **S22/A** | **Ref** | **Kilifi** | **S22/A** |
| Severe  anaemia | P6566 | 0 | 0 | 0 | 0 | - | - | - |
|  | P7103 | 198.2 | 179.8 | 46.7 | 120.3 | 69.8 | 19.2 | 31.8 |
|  | P7114 | - | 4.7 | 3.6 | 2.9 | 36.2 | 2.9 | 19.9 |
|  | P8717 | - | - | - | - | 129.5 | 71.3 | 57.9 |
|  | P8745 | 452.7 | 138.4 | 160.5 | 174.9 | 28.6 | 5.1 | 0 |
|  | P8751 | 0 | 0 | 0 | 0 | 0 | 0 | 0 |
|  | P8792 | 13.2 | 40.4 | 226.8 | 1.9 | - | - | - |
|  | P9200 | 286.9 | 108.4 | 0.7 | 51.4 | - | - | - |
| Cerebral  malaria | P6507 | 428 | 0 | 0 | 0 | 0 | 0 | 0 |
|  | P6566 | 0 | 0 | 0 | 0 | - | - | - |
|  | P6569 | 61.3 | 0 | 0 | 21.6 | - | - | - |
|  | P6610 | 0 | 0 | 0 | 0 | - | - | - |
|  | P6847 | 469.1 | 316.2 | 199.4 | 190.1 | - | - | - |
|  | P7038 | 69.6 | 513.6 | 71.9 | 508.2 | 85.7 | 10.1 | 93.8 |
|  | P7042 | 150.5 | 660.3 | 168.7 | 588.6 | 401.8 | 18.8 | 190.6 |
|  | P7071 | 2605.6 | 1631.2 | 988.1 | 1768.7 | 460.8 | 185.9 | 218.1 |
|  | P7132 | 45.8 | 240.2 | 752.5 | 591.4 | 138.9 | 161.7 | 81.4 |
|  | P7768 | 1341.3 | 256.2 | 150.4 | 30.5 | 42 | 5.8 | 39.8 |
|  | P7942 | 454.7 | 354.2 | 269.8 | 128.9 | - | - | - |
|  | P8039 | 369.6 | 333 | 13.7 | 0 | 55 | 23.5 | 13 |
|  | P8046 | 33.8 | 279 | 0 | 475.8 | 319.4 | 2.5 | 13.4 |
|  | P8067 | 526.2 | 1348 | 935 | 2386.1 | 269.8 | 133.8 | 200 |
|  | P8738 | 1567.8 | 0 | 0 | 0 | 172.2 | 36.2 | 26 |
|  | P8752 | 443.8 | 437.9 | 281.4 | 722.1 | 82.8 | 49.2 | 43.4 |
|  | P8832 | 703.8 | 0 | 0 | 0 | 1.8 | 4.7 | 0 |
|  | P8846 | 255.1 | 72.8 | 19.7 | 0 | 23.5 | 6.1 | 1.4 |
|  | P9208 | 17.8 | 281.4 | 32 | 337.1 | 0 | 0 | 0 |
| Severe  Malaria-Other | P6388 | 514.9 | 521.9 | 267.2 | 295.1 | 94.8 | 110 | 22.8 |
|  | P6405 | 5.8 | 129.5 | 40.6 | 21.1 | 48.1 | 0 | 0 |
|  | P6466 | - | - | - | - | 29.7 | 17.4 | 0 |
|  | P6567 | 1310.8 | 700.5 | 820.8 | 644.7 | 6.2 | 134.9 | 85.4 |
|  | P7055 | 42.3 | 876.0 | 381.9 | 363.1 | 66.9 | 26 | 25 |
|  | P7057 | 34.7 | 355.9 | 200.4 | 185.9 | 125.9 | 112.5 | 70.9 |
|  | P7067 | - | 652.8 | 271.8 | 448.7 | - | - | - |
|  | P7069 | - | 759.4 | 714 | 272.1 | - | - | - |
|  | P7079 | 77 | 103.1 | 1.8 | 5.1 | 19.5 | 0 | 0 |
|  | P7116 | 237.4 | 205.8 | 9 | 80 | 28.2 | 23.1 | 5.4 |
|  | P7355 | 1689.3 | 1269.7 | 1133 | 1087.8 | 332.8 | 271.3 | 300.2 |
|  | P8050 | 0 | 741.4 | 226.4 | 0 | 28.2 | 23.1 | 5.4 |
|  | P8051 | 1027.6 | 537.1 | 260.2 | 1043.5 | 42.7 | 18.4 | 31.1 |
|  | P8091 | 1029.8 | 30.8 | 0 | 0 | 18.5 | 13 | 0 |
|  | P8119 | - | - | - | - | 5.1 | 0 | 5.8 |
|  | P8213 | 366 | 533.2 | 376.1 | 366.4 | - | - | - |
|  | P8302 | - | - | - | - | 93.3 | 50.3 | 27.9 |
|  | P8705 | 21.1 | 0 | 0 | 0 | 0 | 0 | 0 |
|  | P8718 | 0 | 0 | 0 | 0 | 0 | 0 | 0 |
|  | P8739 | 1067.6 | 259.5 | 321.3 | 22.3 | 4.3 | 20 | 0 |
|  | P8746 | 503.9 | 343.4 | 336.2 | 433.2 | 33.6 | 5.4 | 6.5 |
|  | P8791 | 0 | 0 | 0 | 0 | 0 | 0 | 0 |
|  | P8806 | 667.5 | 130.7 | 0 | 0 | - | - | - |
|  | P8817 | 977 | 276.8 | 0 | 0 | 36.2 | 19.5 | 2.2 |
|  | P8824 | 1739.2 | 0 | 0 | 0 | 6.9 | 1.8 | 0 |
|  | P8825 | 537.3 | 0 | 0 | 0 | 5.4 | 0 | 0.7 |
|  | P8987 | 437.8 | 456.8 | 99.2 | 435.7 | - | - | - |
|  | P9009 | 1600.1 | 21.1 | 0 | 83.3 | 1.1 | 0 | 5.5 |
|  | P9197 | 2234.7 | 1788.3 | 1398.3 | 930.7 | - | - | - |
|  | P9230 | 3.8 | 79.3 | 0 | 18.5 | 0 | 0 | 0 |
| Uncomplicated  malaria | P6370 | 319.3 | 466.6 | 486.4 | 443.6 | - | - | - |
|  | P6402 | 94.2 | 145.1 | 147.1 | 145.4 | 14.4 | 0 | 3.4 |
|  | P6403 | 1497.7 | 113.6 | 129.5 | 38.6 | 0 | 0 | 0 |
|  | P6418 | 662.5 | 210.5 | 216 | 57.7 | 0 | 4.3 | 5.8 |
|  | P6441 | 1532.6 | 670.4 | 495.2 | 499.6 | 91.1 | 32.6 | 62.6 |
|  | P6871 | 1004.7 | 813.6 | 661.5 | 298.4 | 121.9 | 108.5 | 87.5 |
|  | P7148 | 93.7 | 201.3 | 134.7 | 112.5 | 138.9 | 26 | 53.5 |
|  | P7855 | 1975.4 | 1308.9 | 1217.3 | 1102.7 | - | - | - |
|  | P8055 | 208 | 208 | 143.8 | 163.3 | 35.1 | 13.4 | 383.4 |
|  | P8114 | 33.5 | 10.5 | 52.3 | 24.9 | - | - | - |
|  | P8615 | 1546.8 | 57.4 | 0 | 0 | 43 | 21.7 | 12.3 |
|  | P8617 | 3784.4 | 657.9 | 0 | 0 | 43.4 | 34.7 | 18.8 |
|  | P8724 | 732.9 | 876.4 | 506.4 | 440.1 | 299.5 | 239.4 | 356.6 |
|  | P8725 | 433.2 | 459.9 | 258.1 | 130 | - | - | - |
|  | P8735 | 10.3 | 414 | 287.9 | 613 | 42.7 | 24.6 | 17 |
|  | P8740 | 537.1 | 199 | 0 | 0 | 34.4 | 2.5 | 5.1 |
|  | P8742 | 771.8 | 397.4 | 0 | 0 | 29.3 | 30.7 | 5.1 |
|  | P8744 | 212.7 | 54.5 | 33.9 | 26.7 | 6.5 | 0 | 2.5 |
|  | P8748 | - | 70.6 | 75 | 69.2 | 104.2 | 20.6 | 70.5 |
|  | P8759 | 3655.8 | 1249 | 809.5 | 1046.7 | 346.9 | 397.5 | 239.1 |
|  | P8760 | 1051.5 | 17.1 | 0 | 0 | 8.3 | 2.9 | 6.1 |
|  | P8761 | 2079.2 | 14.7 | 0 | 0 | 27.1 | 8.7 | 1.8 |
|  | P8762 | 2736.9 | 245.6 | 242.9 | 219.9 | 64.7 | 28.9 | 58.9 |
|  | P8777 | 748.5 | 0 | 0 | 0 | 0 | 0 | 0 |
|  | P8779 | 2258.3 | 0 | 0 | 0 | 0 | 0 | 0 |
|  | P8780 | 2058.8 | 0 | 0 | 0 | 6.9 | 3.6 | 3.9 |
|  | P8781 | 2229.7 | 0 | 0 | 0 | 9.8 | 7.6 | 7.2 |
|  | P8784 | 270.6 | 0 | 0 | 0 | - | - | - |
|  | P8785 | 1231.3 | 594.5 | 128.8 | 53.6 | - | - | - |
|  | P8788 | 641.6 | 0 | 0 | 0 | 0 | 0 | 0 |
|  | P8793 | 2.6 | 15.6 | 0 | 0 | 0 | 0 | 0 |
|  | P8794 | 13.7 | 14.7 | 16.3 | 17.8 | - | - | - |
|  | P8805 | 0 | 0 | 0 | 0 | - | - | - |
|  | P8810 | 78.6 | 53.3 | 11.3 | 396.2 | - | - | - |
|  | P8815 | 613.5 | 247.3 | 0 | 0 | 52.8 | 7.2 | 0 |
|  | P8833 | 1546.8 | 57.4 | 0 | 0 | 3.3 | 5.4 | 11.6 |
|  | P8836 | 1099.3 | 107 | 0 | 0 | - | - | - |
|  | P8848 | 4076.6 | 228.8 | 76.7 | 19 | 32.2 | 17.4 | 15.2 |
|  | P8849 | 1389.1 | 230 | 67.8 | 34.8 | 76.3 | 38.7 | 11.9 |
|  | P8850 | 2064.4 | 437.1 | 67.5 | 8.2 | 28.9 | 6.5 | 7.9 |
|  | P8877 | 32.9 | 11.1 | 0 | 0 | 0 | 0 | 0 |
|  | P8878 | 744.4 | 0 | 0 | 0 | 0 | 0 | 0 |
|  | P8898 | 1333.6 | 20.9 | 13.5 | 0 | 0 | 0 | 0 |
|  | P8932 | 4053.7 | 498.1 | 250.9 | 281.4 | 40.9 | 20.6 | 15.2 |
|  | P8985 | 333.5 | 57.4 | 51.2 | 0 | 0.7 | 0.4 | 1.2 |

Numbers represent number of adherent parasites/ mm^2^

(-) no data available
