# Supplementary material for: Specific Receptor Usage in Plasmodium falciparum Cytoadherence Is Associated with Disease Outcome
Source: PLoS One. 2011 Mar 3;6(3):e14741. doi: 10.1371/journal.pone.0014741 (PMC3048392; doi:10.1371/journal.pone.0014741)
Supplement: Table S3 — Genotype frequencies for ICAM-1, CD36, α-thalassaemia and HbS. Genotype frequencies for patients studied. HbS, haemoglobin S variant (sickle cell genotype). CD36, point mutation from T to G in the CD36 gene at nucleotide position 188 in exon 10. Numbers represent the number of children that fall into each genotype (wild-type, heterozygous and homozygous). Numbers in parenthesis is given as a percentage of respective genotypes. (0.03 MB DOC) [file pone.0014741.s003.doc]

**Table S3. Genotype frequencies for ICAM-1, CD36, α-thalassaemia and HbS**

|  | Genotype Frequencies % | | | |
| --- | --- | --- | --- | --- |
| Genotype | CD36 | ICAM-1 | α-thalassaemia | HbS |
| Wild-Type | 62.7 | 43.2 | 37.2 | 96.8 |
| Heterozygous | 14.7 | 50 | 52.3 | 3.2 |
| Homozygous mutant | 22.7 | 6.8 | 10.5 | 0 |

Genotype frequencies for patients studied.

HbS, haemoglobin S variant (sickle cell genotype). CD36, point mutation from T to G in the CD36 gene at nucleotide position 188 in exon 10. Numbers represent the number of children that fall into each genotype (wild-type, heterozygous and homozygous). Numbers in parenthesis is given as a percentage of respective genotypes.
